# Data for the measurement of serum vitamin D metabolites in childhood acute lymphoblastic leukemia survivors

**DOI:** 10.1016/j.dib.2018.04.030

**Published:** 2018-04-16

**Authors:** E. Delvin, M. Boisvert, M.-A. Lecours, Y. Théorêt, M. Kaufmann, G. Jones, E. Levy

**Affiliations:** a CHU Ste-Justine Research Centre, University of Montréal, Montréal, Canada; b Department of Biochemistry, University of Montréal, Montréal, Canada; c Clinical Pharmacology Unit, Department of Clinical Biochemistry, CHU Ste-Justine, University of Montréal, Montréal, Canada; d Department of Pharmacology, University of Montréal, Montréal, Canada; e Department of Biomedical and Molecular Sciences, Queen's University, Kingston, Canada; f Department of Nutrition, University of Montréal, Montréal, Canada

## Abstract

This article describes data related to a companion research paper entitled “Vitamin D nutritional status and bone turnover biomarkers in childhood acute lymphoblastic leukemia (cALL) survivors.” (Delvin et al., submitted for publication) [1]. Various methods for the measurement of serum 25OHD_3_, the accepted biomarker for assessing vitamin D nutritional status, have been described (Le Goff et al., 2015; Jensen et al., 2016) [2], [3]. This article describes a novel mass spectrometry-QTOF method for the quantification of circulating 25OHD_3_, 3-epi-25OHD_3_ and 24,25(OH)_2_D_3_. It provides the description of the extraction, chromatography and mass spectrometry protocols, a sample of mass spectra obtained from standards and extracted serum, and a comparison with another HPLC-MS/MS (Jensen et al., 2016) [3] method for the measurement of serum concentrations of 25OHD_3_.

**Specifications Table**

**Table:** 

Subject area	*Biology,*
More specific subject area	*Clinical Chemistry*
Type of data	*Tables, figures*
How data was acquired	*Liquid Chromatography coupled to a quadrupole time-of-flight mass spectrometer (Waters UPLC–MS system (Xevo G2 quadrupole time-of-flight))*
Data format	*Mass spectral analysis, analyzed*
Experimental factors	*Extracted serum samples, blank and standards were analyzed by Liquid Chromatography coupled to a quadrupole time-of-flight mass spectrometry.*
Experimental features	*Vitamin D metabolites were quantified after derivatization with 4-[2-(6,7-Dimethoxy-4-methyl-3-oxo-3,4-dihydroquinoxalinyl)ethyl]-1,2,4-triazoline-3,5-dione **(**DMEQ-TAD) by isotope dilution mass spectrometry*
Data source location	*Montréal, Québec, Canada*
Data accessibility	*The data is available with this article only*

**Value of the data**

• The data describes a novel LC/MS-QTOF method for the measurement of serum vitamin D metabolites, providing the possibility of profiling.
• The details given enable other researchers to reproduce this method.
• This technology will be useful for vitamin D profiling in future clinical studies involving vitamin D supplementation.

## 1 Data

The data shared in this article include the description of the extraction, chromatography and mass spectrometry protocols as well sample mass spectra obtained from standards and extracted serum. The validation procedure of the method and results are also described.

### 1.1 Experimental design, materials and methods

The sample preparation method was adapted from Jones et al. [4]. In brief, 300 μL of a blank, consisting of charcoal-stripped plasma (cat. # 1131-00) purchased from Biocell (Rancho Dominguez, CA, USA), sample, calibrator or control in glass tubes were spiked with 75 μL of a mixture of deuterated internal standards (IS) consisting of [25OHD_3_ (26,26,26,27,27,27-d_6_, IS_1_), 25OHD_2_ (26,26,26,27,27,27-d_6_, IS_2_) from Chemaphor Inc., (Ottawa, ON, Can), 3-epi-25OHD_3_ (6,19,19-d_3_, IS_3_) from Sigma-Aldrich Canada (Oakville, ON, Can) and 24,25(OH)_2_D_3_ (26,26,26,27,27,27-d_6_, IS_4_) from Departmento de Quimica Organica, Laboratorio Ignacio Ribas, University of Santiago de Compostela, 15782 Santiago de Compostela, Spain. 100 µL of a 0.1 M HCl was then added, mixed, and samples allowed to sit for 10 min to release vitamin D metabolites from the vitamin D binding protein. Protein precipitation was achieved by adding 450 µL 0.2 M ZnSO_4_, followed by 900 µL of methanol. The solutions are mixed thoroughly and centrifuged. The supernatants were transferred to borosilicate tubes and 2.2 mL of a 1:1 hexane:methyl tert-butyl ether extraction solvent added. Solutions were vortexes for 20 s. Approximately 1.8 mL was transferred to Waters Total Recovery vials and solutions dried under a gentle nitrogen stream. The dried samples were allowed to react for 90 min with DMEQ-TAD by two successive additions of 25 µL of 0.1 mg/mL of DMEQ-TAD in ethyl acetate after which 40 µL of ethanol was added to quench the reaction and destroy excess DMEQ-TAD. Solutions dried under a nitrogen stream and derivatized vitamin D metabolites solubilized with 20 µL of 40% mobile phase A and 60% mobile phase B (described below).

A Waters UPLC system with a Waters BEH phenyl, 2.1 × 50 mm column with 1.7 µm particle preceded by a guard column was used for the chromatographic separations. Mobile phase A and B consisted of 2 mM ammonium acetate/0.1% formic acid in water and methanol, respectively. Initial conditions were 35% phase A and 65% phase B with a 5 min-gradient to reach 90% phase B, followed by a one-minute equilibration time. The flow rate was 300 µL/min, the column temperature was set at 40 °C, and the auto-sampler at 4 °C. A Waters Xevo G2 QTOF was used to detect and quantify the vitamin D metabolites. The instrument was operated in positive mode using the sensitivity mode. Capillary voltage was set at 1.0 kV, cone voltage at 35 V, with a source temperature of 150 °C, a desolvation gas temperature of 650 °C, with a flow rate of 900 L/h. Mass spectra were acquired in the target-enhanced mode with an acquisition time of 1 s. The chromatographic retention times and ionic transition masses are listed in Table 1.

**Table 1:** Chromatographic retention times and extracted mass ions for each vitamin D metabolite.

Vitamin D metabolite	Extracted ion *m/z*	Retention time (min)
25OHD_3_	746.4740 ± 0.1	4.66 ± 0.1
[^6^d_2_]-25OHD_3_	752.5142 ± 0.1	4.63 ± 0.1
3-epi-25OHD_3_	746.4740 ± 0.1	4.45 ± 0.1
[^3^d_2_]-3-epi-25OHD_3_	749.4966 ± 0.1	4.47 ± 0.1
24,25OHD_3_	762.4680 ± 0.1	3.27 ± 0.1
[^6^d_2_]-24,25OHD_3_	768.5040 ± 0.1	3.24 ± 0.1

The analysis was performed in the scan mode from ions 100–1000 *m/z*. The data acquisition time was 1 s in the continuum mode.

**Fig. 1:**
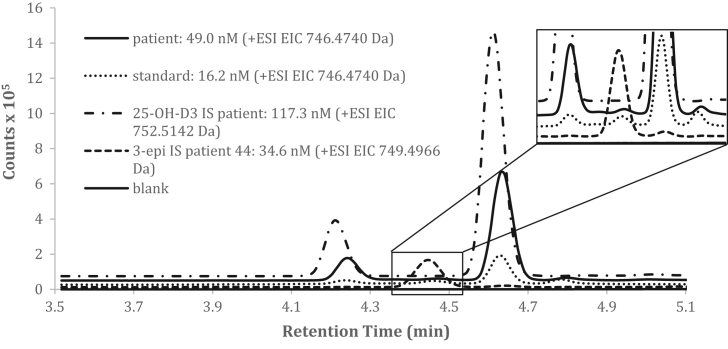
Representative chromatographic profiles of 25-hydroxyvitamin D_3_, 3-epi-25-hydroxyvitamin D_3_ and their respective deuterated internal standards. Superimposed representative chromatographic profiles of a patient sample (), a charcoal-stripped plasma spiked with a 25OHD_3_ standard (….), a charcoal-stripped plasma spiked with the deuterated internal standard [^6^d_2_]-25OHD_3_ (-.-.-), and blank charcoal-stripped plasma () confounded with the X axis. The dotted line (-----) in the inset shows profile for [^3^d_2_]-3-epi-25OHD_3_ in a patient serum extract analysed in the conditions described above.

**Fig. 2:**
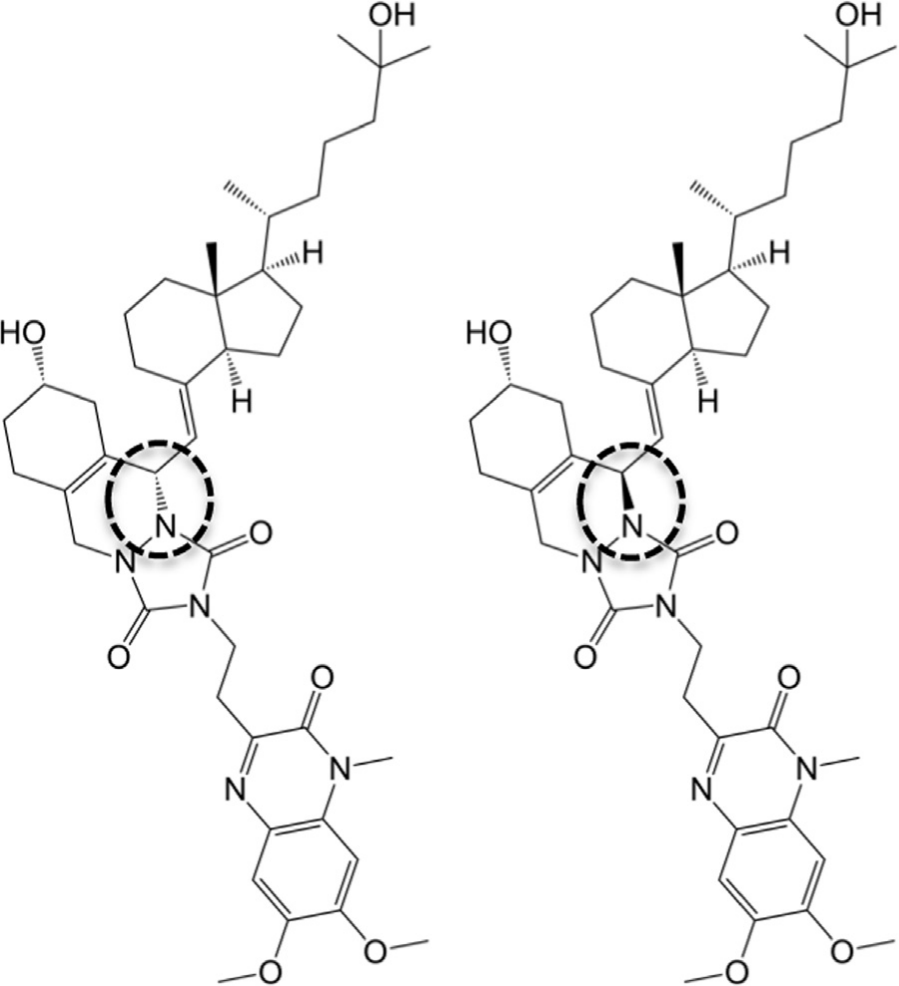
25-hydroxyvitamin D_3_-DMEQ-TAD enantiomers. 25OHD_3_ – DMEQ-TAD: 25-hydroxyvitamin D_3_-4-[2-(6,7-Dimethoxy-4-methyl-3-oxo-3,4-dihydroquinoxalinyl)ethyl]-1,2,4-triazoline-3,5-dione. The dotted circles indicate the chirality centre.

Fig. 1 illustrates superimposed representative chromatographic profiles of a blank charcoal-stripped plasma, a charcoal-stripped plasma spiked with the deuterated internal standards [^6^d_2_]-25OHD_3_, a charcoal-stripped plasma spiked with a standard and a patient sample. The inset in Fig. 1 shows the profile for [^3^d_2_]-3-epi-25OHD_3_ in a patient serum extract analyzed in the conditions described above. Note that the derivatization of the different vitamin D metabolites yielded, for each, 2 DMEQ-TAD enantiomers (R & S) due to an asymmetric carbon (Fig. 2). The major peak was used for quantification.

The method validation was performed with modified CLSI Guidelines [5], [6]. Briefly, the Lower Limit of quantification (LLoQ) was estimated by the serial dilution of the standard solution (*n* = 5 per dilution) and was defined as the concentration at which precision was **≤** 20%. Linearity was evaluated by serially diluting a pool of high 25OHD_3_ concentration samples with charcoal-stripped serum to generate 8 samples of intermediate concentrations that were measured in duplicate. Within assay imprecision was characterized by 5 measurements of a plasma sample pool spiked with 3-epi-25OHD_3_, 25OHD_2_, and 24,25(OH)_2_D_3_. Between-assay imprecision was assessed by analyzing 1 reference sample at 2 different concentrations in each batch over 14 months. Bias was determined by using UTAK vitamin D controls and a DEQAS test sample set.

Table 2A summarizes the performance characteristics of the method. Linear responses were observed up to 462 nM for 25OHD_3_, 158 nM for 25OHD_2_, 148 nM for 3-epi-25OHD_3_ and 149 nM for 24,25(OH)_2_D_3_. The LLOD spanned from 0.1 to 0.3 nM and the LLOQ from 2.0 to 2.5 nM for the 4 vitamin D metabolites. The intra-assay imprecision ranged from respectively 4.2% to 7.0% and the inter-assay imprecision from 8.9% to 10.2% depending on the metabolite measured. The recovery of spiked samples ranged from 92% for 24,25(OH)_2_D_3_ to 118% for 25OHD_3_. As shown in Table 2B, using DEQAS samples, the mean bias for 25OHD_3_ ranged between 6.0 and − 3.1% at 85.7 and 80.0 nM respectively, within the limits set by the Vitamin D Standardization Program [7].

**Table 2A:** Method performance characteristics.

Vitamin D metabolites	25OHD_3_	2425OHD3	3-epi-25OHD_3_	25OHD_2_
LLoD (S/N ≤ 3)	0.3 nM	0.3 nM	0.1 nM	0.3 nM
LLoQ (CV ≤ 20%)	2.5 nM	2.0 nM	2.0 nM	2.5 nM
Intra-assay CV	4.2%	5.1%	4.5%	7.0%
	(70 nM)	(15 nM)	(20 nM)	(40 nM)
Inter-assay CV	7.0%	10.0%	8.9%	10.2%
	(70 nM)	(15 nM)	(20 nM)	(40 nM)
Bias (UTAK vitamin D control)	5%			6%
Linearity r^2^ > 0.99	2.5–462 nM	2.0–149 nM	2.0–148 nM	2.5–158 nM
Spiking % recovery	+ 37.5 nM (118%)	+ 36 nM (92%)	+ 37.5 nM (117%)	+ 37.8 nM (105%)
Carry-over	< LLoD	< LLoD	< LLoD	< LLoD

**Table 2B:** Method bias.

DEQAS controls vitamin D metabolites		25OHD_3_	25OHD_2_	Total (25OHD_3_ + 25OHD_2_)
461	Measured value	56.5 nM	2.7 nM	59.2 nM
	Reference value	55.0 nM	2.1 nM	57.1 nM
	bias	2.7%	28.8	3.7%
462	Measured value	77.5 nM	1.2 nM	78.7 nM
	Reference value	80.0 nM	1.2 nM	81.2 nM
	bias	− 3.1%	0%	− 3.1%
463	Measured value	90.8 nM	1.0 nM	91.8 nM
	Reference value	85.7 nM	0.7 nM	86.4 nM
	bias	6.0%	43%	6.3%
464	Measured value	60.4 nM	1.9 nM	62.3 nM
	Reference value	57.7 nM	2.0 nM	59.7 nM
	bias	4.7%	− 5%	4.4%
465	Measured value	60.0 nM	2.0 nM	62 nM
	Reference value	57.9 nM	2.0 nM	59.9 nM
	bias	3.6%	0%	3.5%

LLoD: Lower Limit of Detection; LLoQ : Lower Limit of Quantification; S/N: Signal/Noise ratio; UTAK : UTAK Laboratories.

**Fig. 3:**
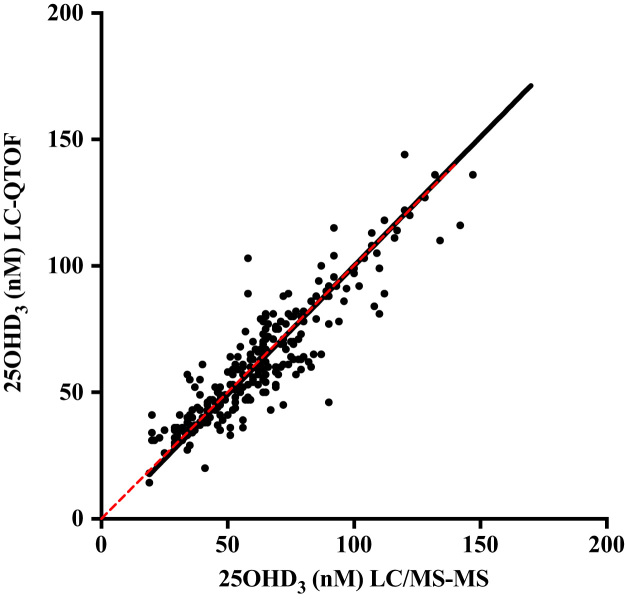
Comparison between the HPLC-MS/QTOF and LC-MS/MS methods for measuring serum 25OHD_3_ concentrations. The Deming linear regression analysis was used for comparing the 2 methods. Dashed line: Line of identity; *n* = 248. Equation: *Y* = 1.02*X–1.91; Slope 95% Confidence Interval (C.I.): 0.957–1.08; *P* = 0.0001.

Fig. 3 is the Deming regression plot comparing serum 25OHD_3_ concentration observed in the 248 serum samples of cALL survivors (1) by the present QTOF method and by HPLC-MS/MS (3). Table 3 lists the 25OHD_3_, 3-epi-25OHD_3_ and 24,25(OH)_2_D_3_ serum concentrations for the same samples.

**Table 3:** 25OHD_3_, 3-epi-25OHD_3_ and 24,25(OH)_2_D_3_ serum concentrations in childhood acute lymphoblastic leukemia survivors.

	LC–MS/MS nM		MS-QTOF nM		
Analyte	Median	2.5–97.5^%iles^	Median	2.5-97.5^%iles^	*P* value
25OHD_3_^b^	60	22–131	57	29–121	= 0.3262
3-epi-25OHD_3_^a^	N/A	N/A	3.1	2.0–12.6	–
24,25(OH)_2_D_3_^b^	N/A	N/A	6.3	2.8–20	–

a 3-epi-25OHD_3_ concentration was ≥ LLoQ in 55 samples.

b *N* = 247.
